# A Low-Cost, Lightweight High-Frequency Ultrasound Transducer with Aluminum Electrodes and 3D-Printed Polymer Housing

**DOI:** 10.3390/bios16090455

**Published:** 2026-08-22

**Authors:** Hyungjung Kim, Woohyun Jin, Do-Kyung Kim, Jaewoo Kim, Jeongwoo Park

**Affiliations:** 1 Department of Biomedical Convergence Science and Technology, Kyungpook National University, Daegu 41566, Republic of Korea; khjung9917@knu.ac.kr (H.K.); woohyun0308@knu.ac.kr (W.J.); 2 Department of Advanced AI Engineering, Kangwon National University, Samcheok 25913, Republic of Korea; dkkim@kangwon.ac.kr; 3 Department of Convergence IT Engineering and Medical Device Innovation Center, Pohang University of Science and Technology (POSTECH), Pohang 37673, Republic of Korea; 4 Department of Advanced Bioconvergence, Kyungpook National University, Daegu 41566, Republic of Korea; 5 Department of Medical and Biological Engineering, Kyungpook National University, Daegu 41944, Republic of Korea; 6 Cell and Matrix Research Institute, Kyungpook National University, Daegu 41944, Republic of Korea

**Keywords:** ultrasound transducer, low-cost transducer, lightweight transducer, high-frequency ultrasound, ultrasound imaging

## Abstract

There is an increasing demand for ultrasound imaging technologies, particularly wearable and portable systems, for continuous physiological monitoring applications. Although some recent flexible ultrasound devices have adopted polymer encapsulations, typical rigid transducer designs still include metal housings and costly electrodes, contributing to increased device weight and fabrication cost. To address these limitations, we developed an aluminum-electrode/3D-printed polymer-housing ultrasound transducer (APUT) utilizing a polyvinylidene fluoride piezoelectric film. Compared to a gold-electrode/metal-housing ultrasound transducer, the APUT material costs and total weight were approximately 66% and 86% lower, respectively. Acoustic evaluation revealed a center frequency of 24.5 MHz and a fractional bandwidth of 60.9%, with axial and lateral resolutions of 51 and 152 μm, respectively. Furthermore, during a 3-h pulsed operation test, the APUT exhibited an initial increase in capacitance followed by a relatively stable response, with no progressive surface-temperature increase detected within the accuracy of the measurement method. Finally, successful ex vivo imaging of chicken breast tissue confirms the APUT’s biomedical applicability, highlighting its potential as a wearable, portable, and disposable ultrasound platform.

## 1. Introduction

The non-invasive nature, real-time imaging capability, and deep penetration depth of ultrasound imaging have led to it being widely used in various fields, including medical imaging [1,2,3,4,5,6,7,8], non-destructive testing [9,10], robotics [11], and underwater exploration [12]. In particular, the growing demand for personalized diagnosis and continuous physiological monitoring has increased the desire for wearable and portable ultrasound systems [13,14,15,16,17]. Specifically, they facilitate the continuous monitoring of blood vessels [18,19], muscles [20,21], and internal organs [22,23,24]. Ultrasound imaging is enabled by ultrasound transducers that convert electrical signals into ultrasonic waves based on the piezoelectric effect and convert pressure variations induced by returning ultrasound waves into electrical signals.

Many conventional ultrasound transducers are fabricated using a ceramic- or single-crystal-based piezoelectric material with high piezoelectric properties and efficient transmission and reception performance such as lead zirconate titanate (PZT) [25,26,27] and lead magnesium niobate–lead titanate (PMN-PT) [8,28,29,30]. However, their large acoustic impedance mismatch with human tissue often requires acoustic matching layers to improve ultrasound transmission and backing layers to attenuate backward-propagating waves and reduce ringing [31,32]. These additional layers can increase the structural complexity, thickness, and fabrication requirements of the transducer. Polyvinylidene fluoride (PVDF) is a representative piezoelectric polymer suitable for compact ultrasound transducers because of its low acoustic impedance and excellent mechanical flexibility [33,34]. In particular, PVDF has a substantially lower acoustic impedance than conventional piezoceramics and a relatively favorable acoustic-impedance match with water and soft tissue, potentially reducing the need for an additional matching layer and enabling structural simplification [35].

Electrode and housing materials are important design considerations for compact PVDF-based ultrasound transducers because they can affect device weight, component cost, electrical performance, structural integrity, and electromagnetic shielding. Gold electrodes offer excellent electrical conductivity and chemical stability, while metallic housings can provide mechanical robustness and electromagnetic shielding [36]. Alternatively, aluminum has good electrical conductivity and a lower raw-material cost than gold, making it a potential electrode material for cost-conscious transducer designs [37]. In addition, 3D printing enables the rapid fabrication of lightweight polymer housings with complex geometries and is advantageous for rapid prototyping [38,39]. However, replacing a metallic housing with a polymer housing may reduce electromagnetic shielding. Therefore, the feasibility of these alternative materials should be evaluated by considering both their potential advantages and performance trade-offs.

Herein, we introduce an aluminum-electrode/3D-printed polymer-housing ultrasound transducer (APUT) based on a PVDF piezoelectric film. As a reference for comparing the two material configurations, a gold-electrode/metal-housing ultrasound transducer (GMUT) was fabricated using the same basic transducer configuration. The APUT was designed to investigate whether aluminum electrodes and a 3D-printed polymer housing could reduce component cost and device weight, respectively, while maintaining suitable acoustic and imaging performance. Owing to the relatively favorable acoustic-impedance match of PVDF with water and soft tissue, both transducers were fabricated without an additional matching layer. The APUT and GMUT were compared in terms of acoustic performance, spatial resolution, device weight, and component cost. Their capacitance and surface temperature were also evaluated during 3 h of pulsed operation. Tissue imaging was conducted to assess the feasibility of the APUT for biomedical imaging applications. This study demonstrates the potential and trade-offs of aluminum electrodes and 3D-printed polymer housings as alternative material choices for compact PVDF-based ultrasound transducers.

## 2. Materials and Methods

### 2.1. Characterization of the Electrode Materials

Commercial PVDF films (PVDF-P0018, PolyK, State College, PA, USA) with a thickness of 18 μm were utilized to fabricate high-frequency ultrasound transducers in the 20 MHz range [40]. To enable ultrasound transmission and reception, we used pre-metallized PVDF films with ~100-nm-thick electrodes on both sides. Specifically, aluminum-coated films were used for the proposed APUT, while gold-coated films were employed for the GMUT (the latter serving as a standard reference ultrasound transducer [41,42]).

Top-view and cross-sectional scanning electron microscopy (SEM; S-4800, Hitachi, Tokyo, Japan) was conducted to analyze the surface morphologies and metal deposition thicknesses of the fabricated metal electrode–PVDF–metal electrode structures. Here, the sheet resistance is explicitly expressed in the conventional unit of ohms per square (Ω/sq) to distinguish it from absolute electrical resistance. The sheet resistance of each electrode material was measured using a four-point probe sheet resistance meter (FPP-400, Dasol Eng., Cheongju, Republic of Korea) to compare their electrical properties. To clarify, these measurements were performed on the rectangular pre-metallized PVDF films before they were cut into 6-mm-diameter circular samples. To evaluate the spatial uniformity of the deposited electrodes, measurements were taken at five different locations on each uncut film sheet, one near the center and four near the corners of the original film. The results were then statistically analyzed.

To evaluate the basic electrical properties of the assembled metal-PVDF-metal structures, the magnitudes and phase angles of their complex electrical impedances were measured in the range of 5–45 MHz using a precision impedance analyzer (Bode 100, OMICRON Lab, Klaus, Austria).

### 2.2. Fabrication of Ultrasound Transducers

**Figure 1:**
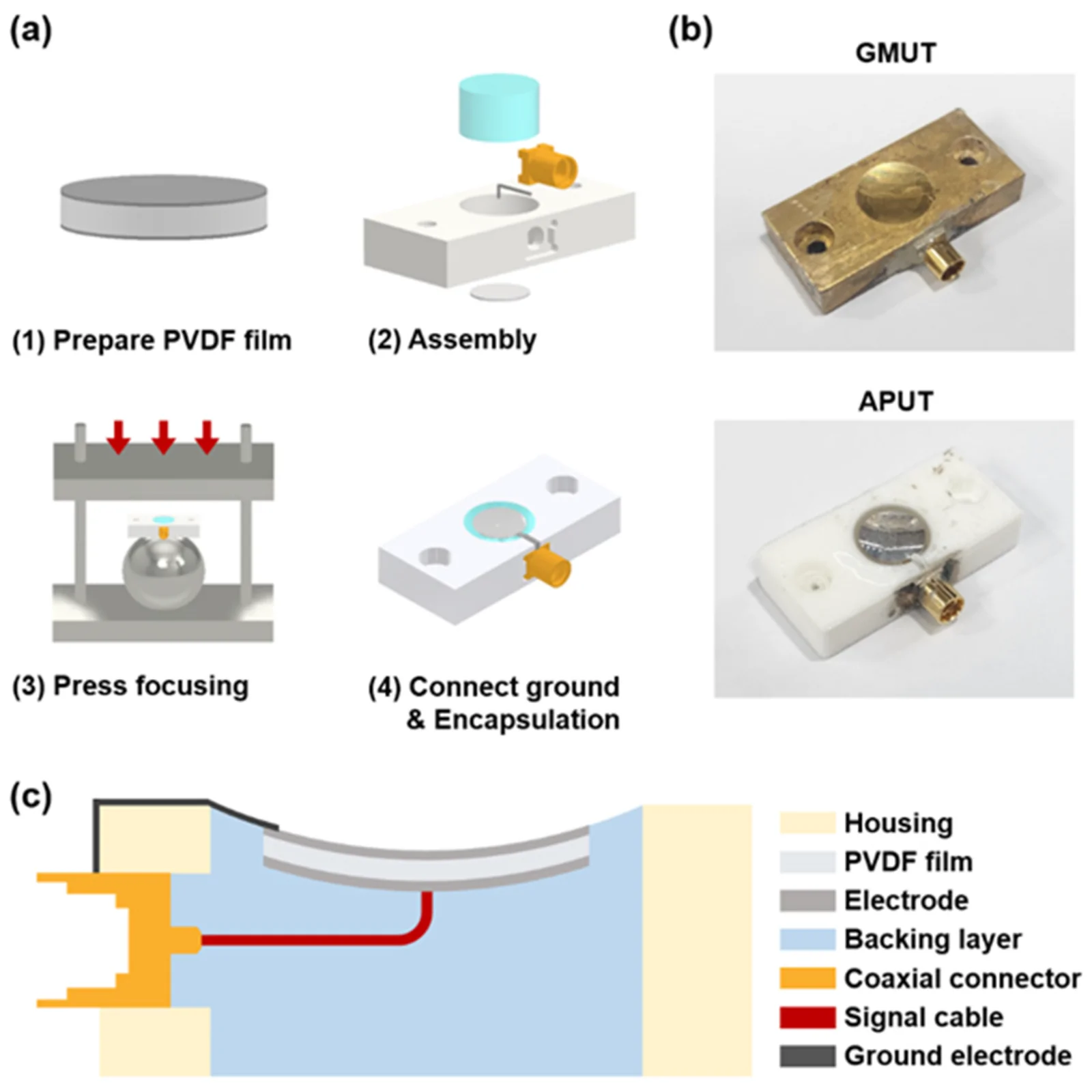
Fabrication and structural overview of the ultrasound transducers. (**a**) Details of the fabrication process for the aluminum-electrode/3D-printed polymer-housing ultrasound transducer (APUT). (**b**) Optical photographs of the fabricated gold-electrode/metal-housing ultrasound transducer (GMUT) and APUT. (**c**) A cross-sectional schematic of the APUT.

First, commercially obtained pre-metallized PVDF films with aluminum or gold electrodes deposited on both sides were cut into 6-mm-diameter circles (Figure 1a(1)). For the transducer body, an acrylonitrile butadiene styrene (ABS) polymer housing fabricated using an FDM-type 3D printer was used for the APUT, while a CNC precision-machined copper housing was utilized for the GMUT. Despite the difference in materials, both housings shared an identical design. The design consisted of a rectangular structure (26 mm × 12 mm × 5 mm) featuring an 8-mm-diameter hole for the PVDF film and screw holes on both sides for mounting.

For each structure, the top electrode of the PVDF film was electrically connected to a signal wire using conductive epoxy (CW2400, Chemtronics, Kennesaw, GA, USA), which was then connected to an MMCX female coaxial connector (Figure 1a(2)). During this process, the PVDF film was aligned such that the center of the film coincided with the center of the hole in the housing. Additionally, the connector was mechanically secured to the housing using the same epoxy. The conductive epoxy was cured in a dry oven at 65 °C for 10 min to ensure rapid curing while preventing both thermal depoling of the PVDF film and structural deformation of the ABS housing. To suppress rearward acoustic radiation and provide vibration damping, degassed non-conductive epoxy (EPO-TEK 301, Epoxy Technology, Billerica, MA, USA) was gently filled into the housing and subsequently cured in a dry oven at 65 °C for 2 h. After curing, the backing layer mechanically fixed the PVDF film to the housing.

After the epoxy was completely cured, a press-focusing process (Figure 1a(3)) was applied to form a geometrically focused single-element transducer [43]. The transducer was heated to 65 °C in a dry oven, and mechanical pressure was applied to the PVDF film using a spherical steel ball with a diameter of 30 mm. The pressing was performed manually using a dedicated jig, and the PVDF film was fully formed into the desired curvature through repeated applications of pressure. After press forming, the transducer was kept in the jig at room temperature until it had sufficiently cooled to maintain the formed geometry. This process formed a concave spherical aperture with a focal length of 15 mm. Following this, ground electrode connections were established for stable ultrasound transmission and reception, and a parylene coating was applied (Figure 1a(4)).

For the GMUT, the copper housing served as the ground structure. To provide an electrical connection between the bottom gold electrode and the housing, a chromium adhesion layer and a gold layer were sequentially deposited by DC sputtering (JWMSS-200XL, Juwon Tech, Ansan, Republic of Korea). Both layers were deposited under an Ar flow rate of 45 sccm and a working pressure of 5.0 mTorr. The chromium layer was deposited at 100 W for approximately 460 s, followed by gold deposition at 80 W for approximately 430 s, to achieve target thicknesses of approximately 50 nm and 100 nm for chromium and gold, respectively. In contrast, for the APUT (which utilizes an insulating polymer housing), a separate grounding pathway was formed from the edge of the bottom aluminum electrode along the surface of the housing to the coaxial connector by applying a thin layer of conductive epoxy.

Finally, to prevent electrical short-circuiting in underwater environments and to protect the surface of the thin PVDF film, a parylene vacuum deposition process (Figure 1a(4)) was performed to form a uniform insulating protective layer on the face of the transducers. During deposition, the coaxial cable connection region was masked to prevent parylene infiltration, while all other exposed surfaces were coated. For this process, 15 g of Parylene C dimer was vaporized and deposited using a commercial parylene deposition system (PDS 2010 Labcoater 2, Specialty Coating Systems, Indianapolis, IN, USA), resulting in a conformal layer with a thickness of approximately 6 μm. This parylene layer provides both an acoustic matching layer and a protective coating. Optical photographs of the fabricated GMUT and APUT are provided in Figure 1b, while a cross-sectional schematic of the APUT is illustrated in Figure 1c.

### 2.3. Ultrasound Imaging Configuration

**Figure 2:**
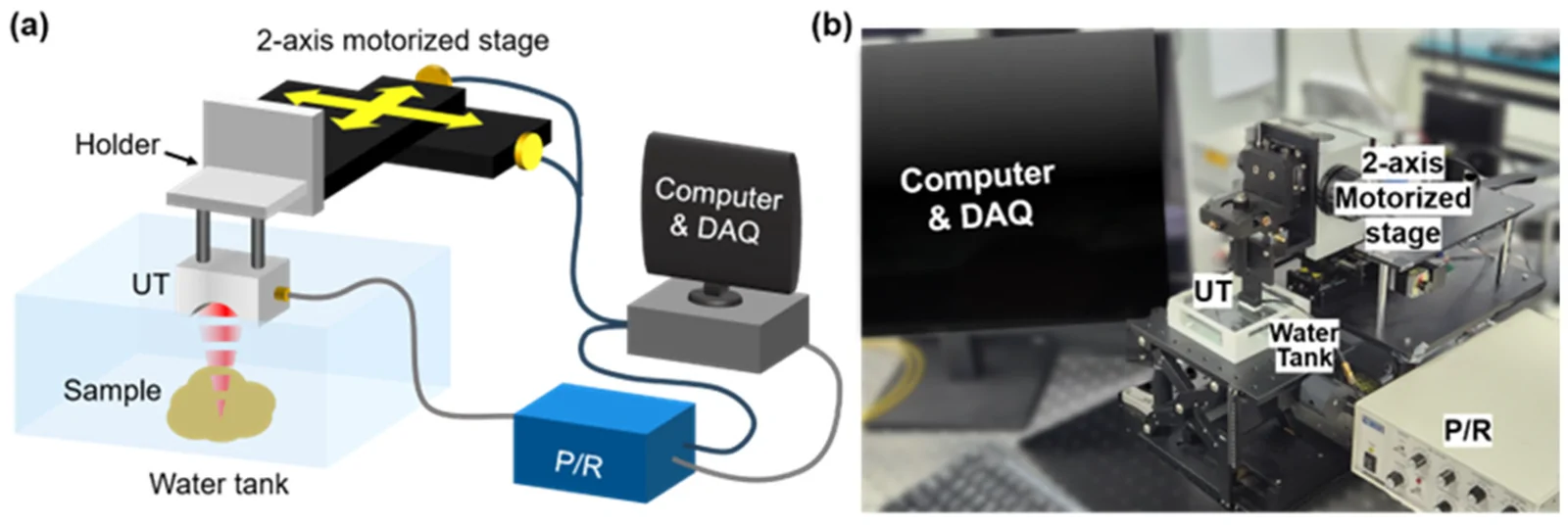
The raster-scanning ultrasound imaging system using the pulse-echo principle. (**a**) A schematic of the system. (**b**) A photograph of the real-world setup. UT, ultrasound transducer; P/R, pulser/receiver; and DAQ, data acquisition board.

To examine the ultrasound imaging performances of the fabricated APUT and GMUT, a raster-scanning ultrasound imaging system was constructed, as shown in the schematic (Figure 2a) and the photograph of the real-world setup (Figure 2b). The transducers were mounted on a 2-axis motorized stage (L-509, Physik Instrumente, Karlsruhe, Germany) to enable 2D mechanical scanning over the imaging plane.

System synchronization was achieved using a multifunction data acquisition (DAQ) board (PCIe-6321, National Instruments, Austin, TX, USA) installed in the control computer. At each scan position, the control software generated trigger signals through the DAQ board to coordinate the motorized stage, pulser/receiver (DPR300, JSR Ultrasonics, Pittsford, NY, USA), and high-speed waveform digitizer (ATS9352, Alazar Technologies, Pointe-Claire, QC, Canada). The echo signals reflected from the sample were amplified by the pulser/receiver and digitized by the waveform digitizer. The digitized ultrasound signals were subsequently processed and visualized using MATLAB R2025a (MathWorks, Natick, MA, USA).

### 2.4. Signal-to-Noise Ratio Calculation

To quantitatively compare the signal quality of the pulse-echo responses, the signal-to-noise ratio (SNR) was calculated from the acquired radio-frequency (RF) data. The signals were first processed using a 5–50 MHz bandpass filter to suppress out-of-band noise. The SNR was then computed using the standard voltage ratio formula:

(1) $$SNR\;(dB)\;=\;20{\;\log}_{10}(\frac{V_{signal}}{V_{noise}})$$

where *V*_signal_ is the peak voltage of the echo envelope obtained using the Hilbert transform, and *V*_noise_ is the root-mean-square (RMS) voltage of a pre-echo background region.

If an RF waveform is obtained by averaging *N* repeated acquisitions, the measured SNR includes the improvement resulting from signal averaging. Assuming independent random noise across acquisitions, averaging reduces the RMS noise voltage by a factor of $\sqrt{N}$ without changing the echo amplitude [44,45]. Therefore, the SNR corresponding to a single acquisition was estimated as:

(2) $$SNR_{single}\;(dB)\;=\;SNR_{avg}\;(dB)\;-\;10\;\log_{10}(N)$$

where SNR_avg_ is the SNR calculated from the averaged RF waveform, SNR_single_ is the estimated SNR of a single acquisition, and *N* is the number of averaged acquisitions. For the pulse-echo measurements, 16 acquisitions were averaged (*N* = 16). Therefore, the averaging gain of 12.0 dB was subtracted from the SNR measured from the averaged RF waveform to estimate the single-acquisition SNR.

### 2.5. Theoretical Axial and Lateral Resolution Calculations

Evaluating the theoretical resolution and comparing it with measured values is essential to validate the successful fabrication and overall acoustic performance of the ultrasound transducer [46]. The theoretical limit of the axial resolution can be calculated using the following equation [47]:

(3) $$R_{axial}\;=\;\frac{c}{2∆f}\;=\;\frac{c}{2\;(f_{c}\cdot FBW)}$$

where *c* is the speed of sound in degassed water (1480 m/s), Δf is the −6 dB bandwidth, *f*_c_ is the measured center frequency, and fractional bandwidth (FBW) is defined as $\Delta f/f_{c}$.

The theoretical limit of the lateral resolution is calculated using the following equation [47]:

(4) $$R_{lateral}=\frac{c\cdot F_{\#}}{f_{c}}=\lambda \cdot F_{\#}=\lambda \cdot \frac{F}{D}$$

where *F*_#_ represents the *f*-number, defined as the ratio of focal length to the aperture size of the ultrasound transducer. λ is the wavelength, *F* is the focal length (15 mm), and *D* is the effective aperture (5.96 mm). Regarding the effective aperture, the PVDF film was cut into a 6-mm-diameter circle based on the impedance matching design. During the press-focusing process, the film was deformed into a curved surface using a spherical ball with a diameter of 30 mm over the central hole of the housing with a diameter of 8 mm. From this geometrical configuration, the final effective diameter (*D*) of the transducer was calculated to be approximately 5.96 mm.

### 2.6. Preparation of the Biological Tissue Specimen

To evaluate the practical imaging performance of the fabricated transducers, an ex vivo biological tissue specimen was prepared using fresh chicken breast due to it providing a biological matrix with acoustic scattering and attenuation characteristics relevant to ultrasound imaging [48]. The tissue specimen was prepared by thinly slicing the chicken breast and carefully stacking the layers to achieve a total thickness of approximately 3 mm. To prevent the entrapment of air bubbles, a thin layer of degassed acoustic gel was applied between each tissue layer, and the stacked layers were gently compressed. Within this stacked tissue matrix, three distinct imaging targets were embedded at specific depths to comprehensively evaluate both spatial resolution and structural contrast. First, a layer of native fascia, naturally extracted from the chicken breast, was embedded at a depth of 0.5 mm to assess the transducer’s ability to differentiate subtle acoustic impedance mismatches. Second, a 23-gauge stainless steel needle with an outer diameter of 0.64 mm was inserted at a depth of 2.0 mm. Finally, two 40-μm-thick wires were positioned at depths of 0.5 and 1.0 mm, respectively. During the imaging procedure, the entire prepared specimen was submerged in a degassed water tank.

### 2.7. 3-h Pulsed Operation Test

The electrical and thermal responses of the APUT during repeated pulsed operation were compared with those of the metal-housed GMUT. Both transducers were operated in air for 180 min using a pulser/receiver at a pulse repetition frequency (PRF) of 1 kHz. Testing in air provided a reduced acoustic-loading condition without water coupling.

Capacitance and surface temperature were monitored as representative electrical and thermal parameters, respectively. Measurements were obtained before operation and subsequently at 10 min intervals throughout the 180 min test, resulting in 19 measurements for each transducer. Capacitance was measured at a test frequency of 100 kHz in series capacitance mode using a handheld LCR meter (U1733C, Keysight Technologies, Santa Rosa, CA, USA). Before the experiment, open and short calibrations were performed to compensate for the residual impedance and stray capacitance of the test leads. The meter remained powered on, and the measurement frequency, equivalent-circuit mode, and connection configuration were maintained throughout the 3 h test. The displayed capacitance resolution was 0.1 pF. Under the applied frequency and measurement range, the manufacturer-specified accuracy is ± (2.0% of the reading + 10 counts), corresponding to approximately ±3.4–3.9 pF over the measured capacitance range.

Surface temperature was measured using a non-contact infrared thermometer (VICTOR 303B, Shenzhen Victor Hi-Tech, Shenzhen, China), which has a display resolution of 0.1 °C and a manufacturer-specified measurement accuracy of ±3.0 °C within the measured temperature range of 0–25 °C. Each capacitance and temperature data point represents a single measurement obtained at the corresponding time point.

## 3. Results

### 3.1. Morphological and Electrical Characteristics of the Electrode Materials

**Figure 3:**
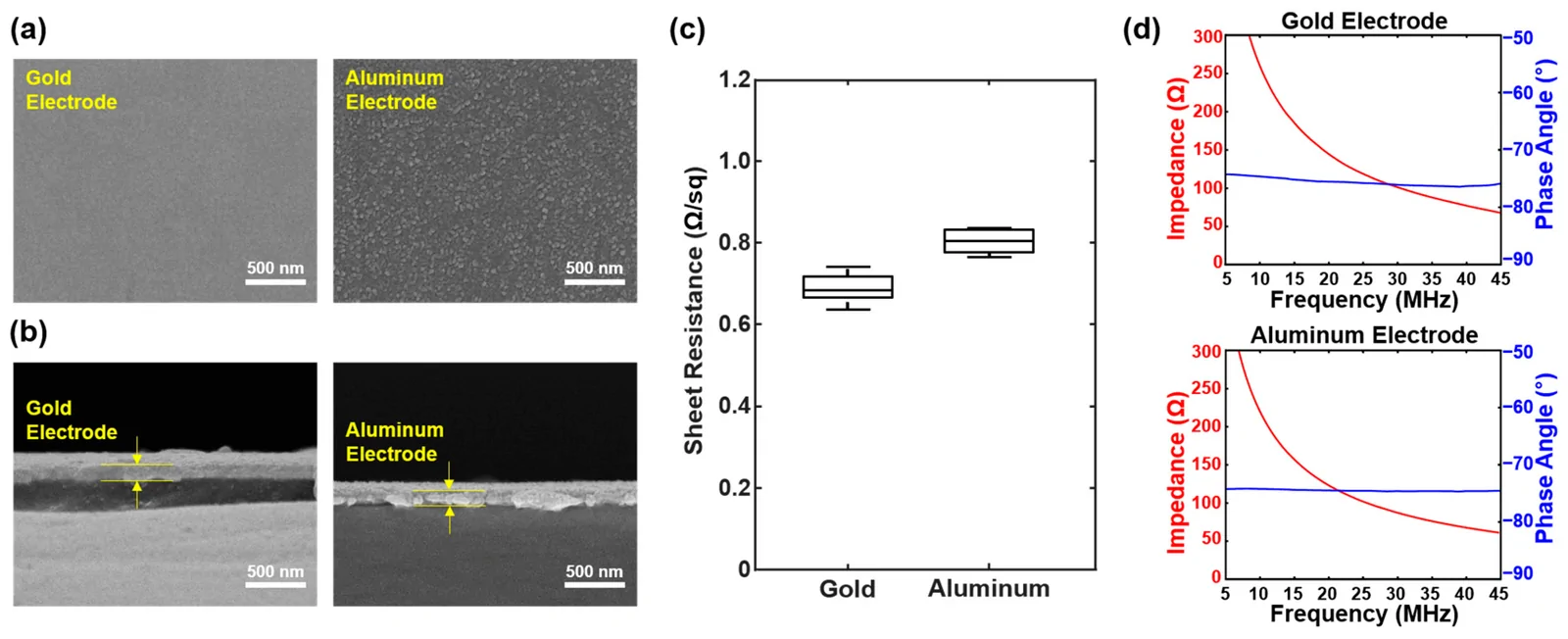
Structural and electrical characterization of the gold and aluminum electrodes deposited on polyvinylidene fluoride (PVDF) films. (**a**) Top-view and (**b**) cross-sectional view scanning electron microscopy images of the sputtered electrodes on the PVDF films, indicating deposition thicknesses of approximately 99 and 110 nm for the gold and aluminum electrodes, respectively. (**c**) Boxplot of the sheet resistances of the fabricated electrode materials, measured on the rectangular pre-metallized PVDF films before cutting into 6-mm-diameter circular samples. For each material, measurements were taken at five locations on the original uncut film sheet, one near the center and four near the corners. The central mark indicates the median, and the bottom and top edges of the box indicate the 25th and 75th percentiles, respectively. The whiskers extend to the minimum and maximum data points. (**d**) Electrical impedance magnitudes and phase angles of the fabricated gold and aluminum electrode–PVDF–electrode structures.

The top-view SEM images of the gold and aluminum electrodes on PVDF films in Figure 3a reveal the similar surface morphologies of the electrode materials, while the cross-sectional images in Figure 3b indicate that the actual thicknesses of the gold and aluminum electrodes on the PVDF films were approximately 99 and 110 nm, respectively. Meanwhile, the sheet resistances of the gold and aluminum electrodes were 0.69 ± 0.04 Ω/sq and 0.80 ± 0.03 Ω/sq, respectively (Figure 3c). As shown in Figure 3d, the aluminum-electrode-based structure exhibited impedance magnitude and phase angle responses comparable to those of the gold-electrode-based structure.

### 3.2. Cost and Weight Comparison of the Ultrasound Transducers

To validate the feasibility of the proposed APUT, a comparison of the material costs and total weights of the APUT and GMUT is summarized in Table 1. To clarify these calculations, the material cost of each component was determined by multiplying its unit cost by the actual amount used for a single transducer. For the APUT housing, the 3D-printing cost was estimated based on an outsourcing quotation rather than solely on the raw material cost to ensure a fair comparison. The signal wire and conductive epoxy used to secure it were excluded from Table 1 because their amounts and costs were negligible. Because the ground electrodes of the two transducers were fabricated using fundamentally different processes, a direct one-to-one comparison of their costs was difficult. Specifically, fabrication of the GMUT ground electrode requires metal thin-film deposition, whereas fabrication of the APUT ground electrode does not. Therefore, the cost of the GMUT ground electrode was estimated from a batch-processing quotation based on the maximum number of transducers that could be fabricated in a single deposition run. In contrast, the cost of the APUT ground electrode was calculated from the actual amount of material used for a single transducer. The material costs reported in Table 1 may vary depending on currency exchange rates.

The total cost of the APUT was 6.74 USD, whereas that of the GMUT was 19.57 USD, corresponding to an approximately 66% cost reduction for the APUT. This reduction primarily resulted from the lower costs of the housing and electrode-related components. In particular, the APUT housing cost was 4.22 USD, which was substantially lower than the GMUT housing cost of 11.63 USD. Overall, the proposed APUT showed a lower estimated fabrication cost than the GMUT owing to the use of lower-cost alternatives to the precision-machined copper housing and electrode-related components.

Moreover, the total weight of the APUT was 1.6 g, which was substantially lower than that of the GMUT (11.3 g), corresponding to an approximately 86% reduction. This large difference is mainly attributed to the lower density of the polymer-based housing material used for the APUT compared with the copper housing used for the GMUT. Specifically, the density of the ABS filament used for the APUT housing was approximately 1.05 g/cm^3^, whereas that of copper used for the GMUT housing was approximately 8.9 g/cm^3^. This difference in density is a major factor contributing to the markedly lower weight of the APUT. It should be noted that the total weights presented in Table 1 were measured after the final fabrication was completed.

### 3.3. Pulse-Echo Response and Frequency Characteristics

**Figure 4:**
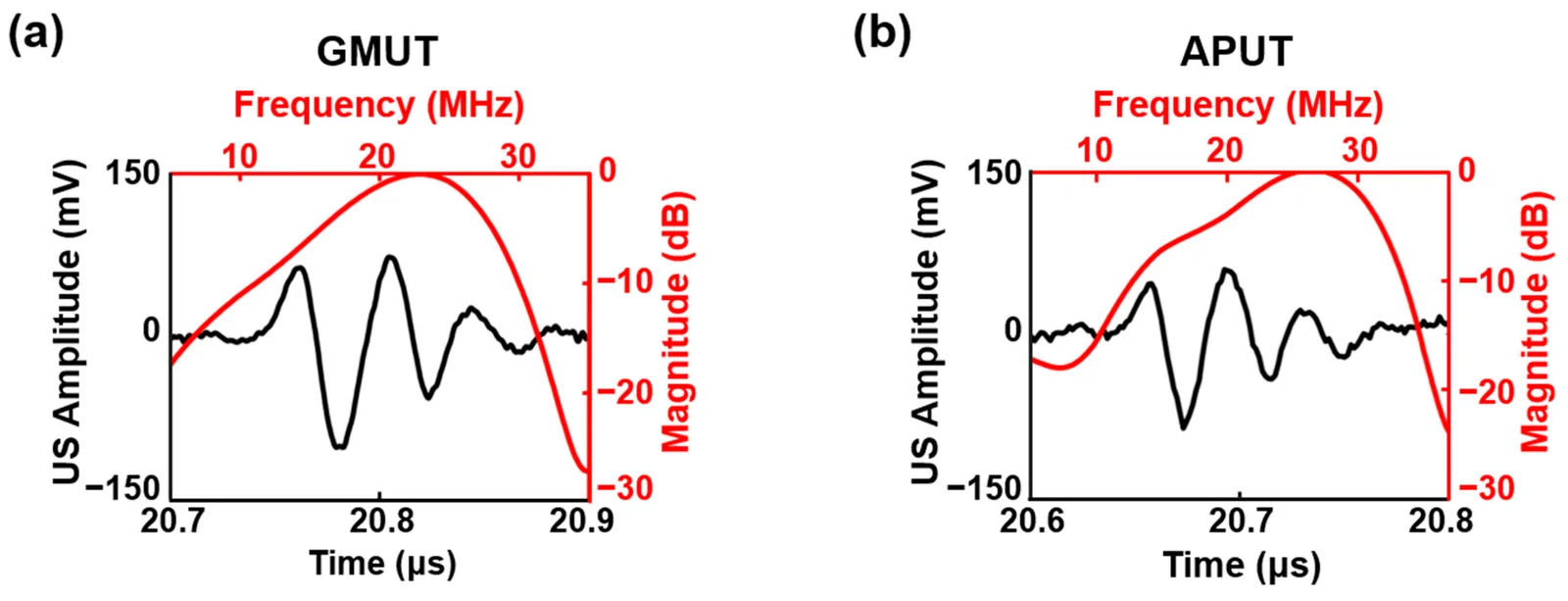
Measured pulse-echo response waveforms in the time domain (black lines) and corresponding frequency spectra (red lines) for (**a**) the GMUT and (**b**) the APUT.

The acoustic performances of the fabricated APUT and GMUT were evaluated by analyzing their pulse-echo responses and frequency spectra (Figure 4). A quartz block was used as the reflector target for pulse-echo measurements. In the time-domain pulse-echo signals, the main echo peaks appeared at approximately 20.7 μs for the APUT and 20.8 μs for the GMUT. Considering the round-trip propagation time in water, these echo arrival times correspond to a reflector distance of approximately 15 mm, which is consistent with the designed geometric focal length. The center frequency and −6 dB FBW were calculated from the frequency spectra obtained using fast Fourier-transform analysis. The center frequencies for the APUT and GMUT were 24.5 and 21.8 MHz, respectively. The −6 dB FBW values of the APUT and GMUT were 60.9% and 62.6%, respectively, thereby indicating nearly identical broadband acoustic responses. The peak-to-peak voltages of the received echo signals were 146.8 mV for the APUT and 175.2 mV for the GMUT under the same excitation and reception conditions. Based on this analysis, the SNRs calculated from the RF waveforms averaged over 16 acquisitions using Equation (1) were 25.2 dB for the APUT and 32.6 dB for the GMUT. After correcting for the averaging gain of 12.0 dB using Equation (2), the estimated single-acquisition SNRs were 13.2 dB for the APUT and 20.5 dB for the GMUT. Thus, the GMUT exhibited a 7.3 dB higher estimated single-acquisition SNR than the APUT under the same measurement conditions.

### 3.4. Spatial Resolution Measurement

**Figure 5:**
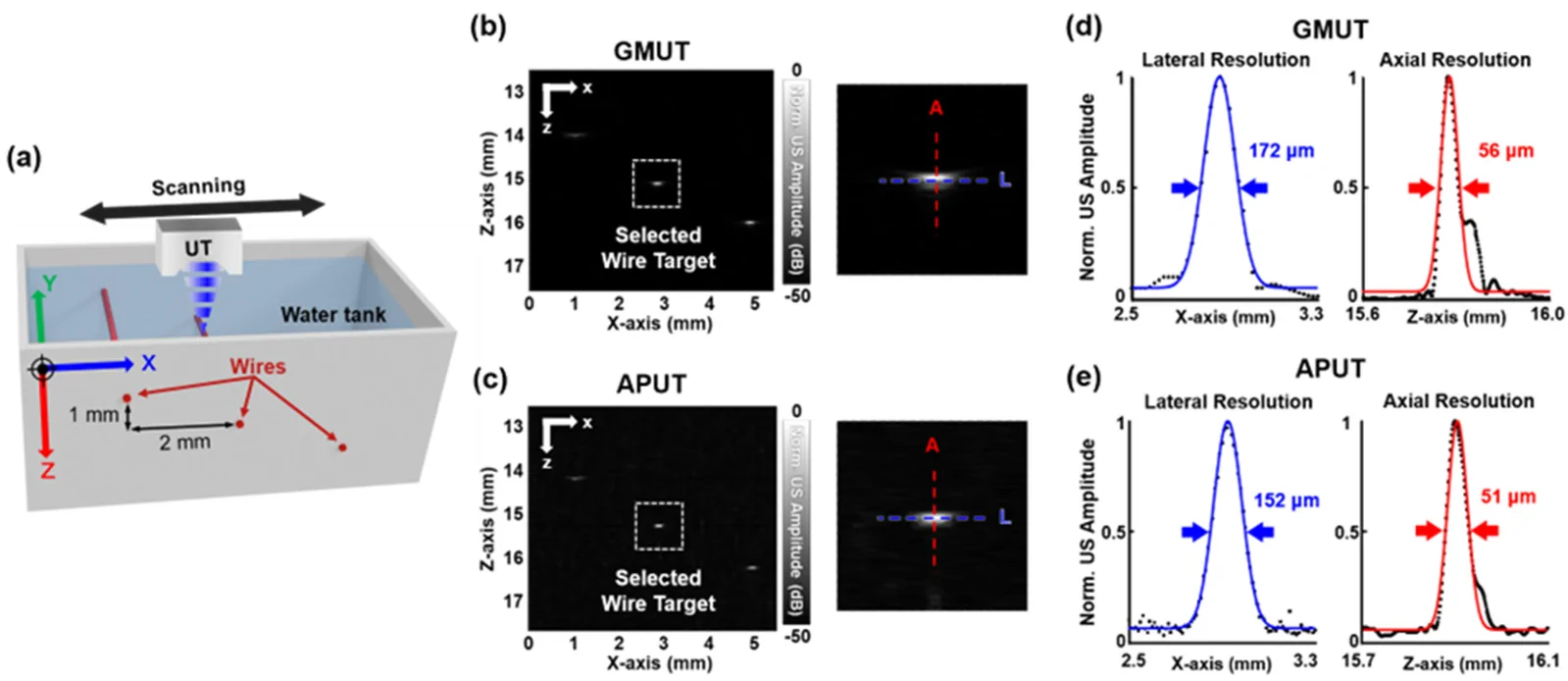
Spatial resolution analysis using a wire phantom. (**a**) Schematic of the wire phantom consisting of multiple copper wires immersed in a water tank with a lateral spacing of 2 mm and a depth spacing of 1 mm. The X-, Y-, and Z-axes denote the lateral, elevational, and axial directions, respectively. Ultrasound B-scan images obtained using (**b**) the GMUT and (**c**) the APUT. The white dashed boxes indicate the regions containing the selected wire target, which are shown as magnified views in the right panels. In the magnified images, the blue horizontal dashed line labeled “L” and the red vertical dashed line labeled “A” pass through the maximum-amplitude pixel and indicate the lateral and axial profile extraction directions, respectively. Normalized lateral and axial line spread function data and their corresponding Gaussian fits for (**d**) the GMUT and (**e**) the APUT. The individual black points represent the normalized profile samples, whereas the solid curves represent the Gaussian fits used to determine the experimental full width at half maximum of each profile.

The spatial resolutions of the fabricated transducers were evaluated using a wire phantom composed of multiple copper wires immersed in a water tank, with a lateral spacing of 2 mm and a depth spacing of 1 mm, as illustrated in Figure 5a. To facilitate comparison between the B-scan images and the extracted profiles, an X–Y–Z coordinate system was added to Figure 5a, where the X-axis denotes the lateral scanning direction and the Z-axis denotes the axial depth direction. The GMUT and APUT were positioned above the water tank and mechanically scanned to acquire ultrasound B-scan images of the wire phantom, as shown in Figure 5b,c. The B-scan images used for the resolution analysis were reconstructed directly from the acquired raw data. To improve signal stability and suppress random background noise, 10 adjacent B-scans acquired from the same region were averaged to generate a representative B-scan image for each transducer.

To quantitatively evaluate the spatial resolution, the central wire target located near the focal length of the transducers, at an approximate depth of 15 mm, was selected for analysis. The regions containing the selected wire target are indicated by the white dashed boxes in Figure 5b,c and are presented as magnified images. Within the response of the selected wire target, the pixel exhibiting the maximum signal amplitude was defined as the center point for profile extraction. A lateral amplitude profile was extracted along the X-direction through this center point, whereas an axial amplitude profile was extracted along the Z-direction through the same point. In the magnified images, the blue horizontal dashed line labeled “L” and the red vertical dashed line labeled “A” indicate the lateral and axial profile extraction directions, respectively.

Each extracted profile was independently normalized by its maximum amplitude such that the peak value was set to 1. The normalized profile samples, representing the discrete line spread function (LSF) data, are plotted as individual points, whereas the solid curves represent Gaussian fits to the LSF data. The experimental lateral and axial resolutions were defined as the full widths at half maximum (FWHMs) of the corresponding fitted LSFs, measured as the distance between the two positions on opposite sides of the peak at which the fitted amplitude reached half of its maximum value. The resulting normalized data points, Gaussian-fitted LSFs, and corresponding FWHM measurements are presented in Figure 5d for the GMUT and Figure 5e for the APUT.

For comparison, the theoretical axial and lateral resolutions were calculated using Equations (3) and (4), respectively, as described in Section 2.5, based on the measured center frequency, −6 dB FBW, focal length, and effective aperture of each transducer. The theoretical lateral resolutions of the GMUT and APUT were 171 and 152 μm, respectively, whereas the experimentally measured lateral resolutions were 172 and 152 μm, respectively. Likewise, the theoretical axial resolutions of the GMUT and APUT were 54 and 49 μm, respectively, whereas the experimentally measured axial resolutions were 56 and 51 μm, respectively. Overall, the experimental estimates showed reasonable agreement with the corresponding theoretical predictions.

### 3.5. Ex Vivo Biological Tissue Imaging

**Figure 6:**
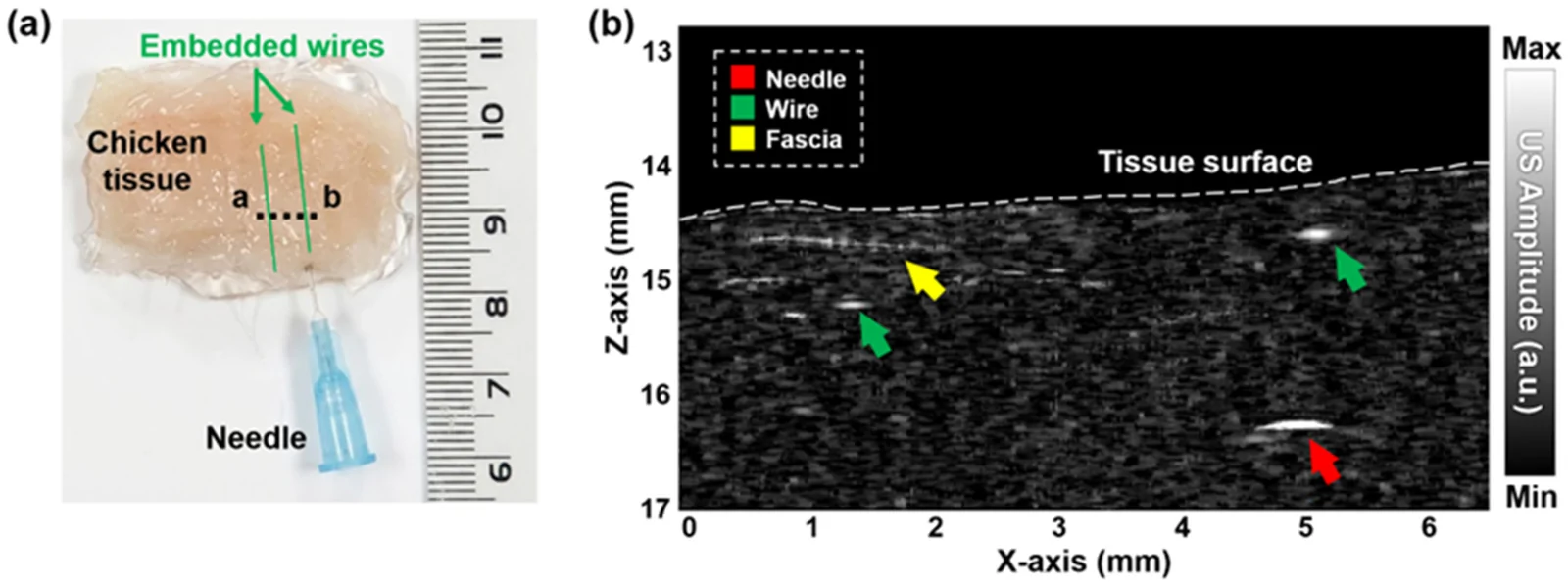
Chicken breast tissue imaging using the APUT. (**a**) A photograph of the chicken breast imaging setup and (**b**) acquired ultrasound B-scan image from the tissue surface to a depth of approximately 3 mm.

To further evaluate the ultrasound imaging capability of the APUT in a biological tissue environment, B-scan imaging was performed on chicken breast tissue (Figure 6a), into which a native fascia, a metallic needle, and two embedded wires were placed to evaluate the visualization of tissue-embedded structures. The APUT successfully acquired ultrasound B-scan images from the tissue surface to a depth of approximately 3 mm (Figure 6b). In the reconstructed B-scan image, the metallic needle (indicated by the red arrow) generated the strongest echo signal due to its high acoustic impedance mismatch with the surrounding soft tissue. The embedded wires (indicated by the green arrows) were also clearly visible as localized high-contrast structures within the tissue. In contrast, although the native fascia (indicated by the yellow arrow) produced relatively lower echo intensity than the metallic targets, it was still distinguishable from the surrounding chicken breast tissue. These results provide preliminary evidence that the proposed APUT can acquire meaningful ultrasound images from a biological tissue context, thereby supporting its practicability as a low-cost, lightweight, wearable-compatible, and potentially disposable ultrasound platform.

### 3.6. 3-h Operational Test

**Figure 7:**
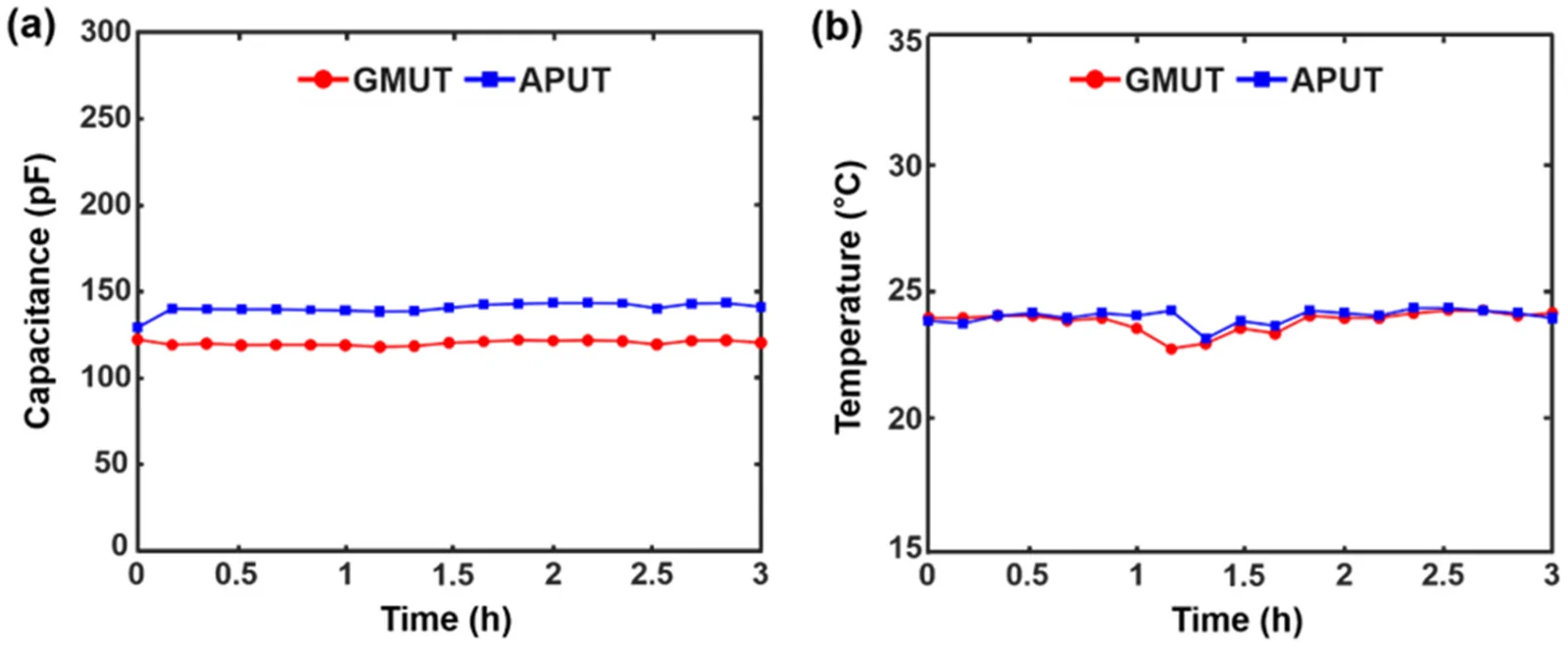
Electrical and thermal responses of the fabricated transducers during 3 h of pulsed operation in air at a PRF of 1 kHz. (**a**) Capacitance variations and (**b**) surface temperature profiles of the GMUT and APUT, recorded at 10 min intervals.

To compare their electrical and thermal responses during pulsed operation, the APUT and metal-housed GMUT were operated in air for 3 h at a PRF of 1 kHz. Their capacitances and surface temperatures were recorded at 10 min intervals, resulting in 19 measurements for each transducer (Figure 7).

As shown in Figure 7a, the capacitance of the GMUT exhibited only minor fluctuations without a progressive increasing or decreasing trend over the 3 h period. In contrast, the capacitance of the APUT increased from 128.7 to 139.9 pF during the first 10 min, corresponding to an increase of approximately 8.7%. After this initial change, the APUT capacitance remained within a relatively narrow range without a further progressive increase.

As shown in Figure 7b, the recorded surface temperatures ranged from 22.8 to 24.3 °C for the GMUT and from 23.2 to 24.4 °C for the APUT. Although point-to-point variations were observed, neither transducer showed a progressive increase in surface temperature during the 3 h test. Because each time point represented a single measurement and the observed variations were smaller than the specified accuracy of the infrared thermometer (±3.0 °C at 0–25 °C), the differences between individual temperature measurements were not considered quantitatively significant.

Overall, under the investigated 3 h pulsed-operation condition in air, the APUT exhibited an initial increase in capacitance followed by a relatively stable response, while no progressive surface-temperature increase was detected within the accuracy of the measurement method.

## 4. Discussion

We developed a low-cost and lightweight high-frequency ultrasound transducer by integrating aluminum electrodes and a 3D-printed polymer housing with a PVDF piezoelectric film. Under the cost assumptions specified in Table 1, the estimated component-level costs of the APUT and reference GMUT were 6.74 and 19.57 USD, respectively, corresponding to an estimated reduction of approximately 66%. The measured weights of the fully assembled APUT and GMUT were 1.6 and 11.3 g, respectively, corresponding to an approximately 86% reduction. The APUT also retained a broadband acoustic response and high-frequency imaging capability, although its lower echo amplitude and SNR indicate that further performance optimization is required. These findings support the feasibility of aluminum electrodes and a 3D-printed polymer housing as alternative material choices for compact PVDF-based ultrasound transducers.

The APUT exhibited a −6 dB FBW nearly equivalent to that of the GMUT, together with a higher center frequency and a lower echo voltage. The higher center frequency may be partly associated with the lower mass loading of the aluminum electrode on the thin PVDF film [49,50]. The lower echo voltage may be related to differences in electrode conductivity, sheet resistance, electrode–film interfaces, grounding, and transducer assembly [51,52]. Because these factors were not varied independently, their individual contributions to the observed acoustic differences could not be determined in the present study.

The APUT exhibited smaller measured axial and lateral FWHM values than the GMUT, indicating finer spatial resolution under the investigated conditions. These smaller resolution distances may be partly attributable to the higher center frequency of the APUT, consistent with the theoretical relationships described in Section 2.5. However, bandwidth, aperture geometry, focusing, acoustic-field characteristics, and the profile-extraction procedure may also affect the measured resolution. Therefore, the principal significance of the APUT lies not simply in its smaller measured resolution values, but in its ability to retain high-frequency imaging performance using a lighter and lower-cost material configuration.

One possible factor contributing to the higher measured noise of the APUT is the absence of the electromagnetic shielding inherently provided by a metallic enclosure. In an additional pulse–echo comparison performed under nominally identical conditions within the same measurement session, applying a grounded copper foil layer to the plastic housing reduced the RMS noise voltage of the APUT from 9.7 to 5.1 mV and increased its SNR from 11.6 to 17.1 dB (Figure S1). However, its noise performance did not reach that of the GMUT. Because no controlled electromagnetic-interference source was applied and the transducers also differed in their electrode and grounding configurations, these findings indicate reduced measured noise under the tested laboratory conditions rather than quantitative housing shielding effectiveness. Further optimization of the shielding and grounding design, together with standardized electromagnetic-compatibility testing under clinically representative conditions, will therefore be required before clinical implementation.

The successful identification of the embedded targets in the layered ex vivo chicken-breast tissue specimen further supports the feasibility of using the APUT for ultrasound imaging in a biological-tissue environment. However, the prepared specimen, which contained stacked tissue layers, acoustic gel, and artificially embedded targets, did not reproduce the full acoustic and physiological complexity of in vivo tissue. Evaluation using well-characterized tissue-mimicking phantoms, followed by in vivo validation, will therefore be required to establish the biomedical imaging performance of the APUT.

During the 3-h pulsed-operation test in air at a PRF of 1 kHz, pulsed excitation was briefly stopped at 10-min intervals to permit capacitance measurement using the LCR meter. The APUT capacitance increased from 128.7 to 139.9 pF during the first 10 min, corresponding to an increase of approximately 8.7%, and subsequently showed no clear progressive increasing or decreasing trend. This initial change may be associated with short-term electrical equilibration or interfacial effects involving the aluminum electrode, including contributions from the pre-existing native aluminum oxide layer and interfacial polarization under repeated pulsed excitation [53,54,55,56]. However, these explanations remain speculative because the dielectric properties and electrode–film interface were not independently examined. In addition, because the transducers were manually disconnected and reconnected at each measurement time point and only one capacitance value was recorded, small point-to-point variations may include variability arising from repeated electrical connections. Further dielectric, interfacial, and repeated-measurement analyses are required to determine the origin and reproducibility of the initial capacitance change.

The indicated surface temperatures ranged from 22.8 to 24.3 °C for the GMUT and from 23.2 to 24.4 °C for the APUT, with no evident progressive increase during the investigated 3-h period. However, each time point represented a single measurement, and the observed variations were smaller than the manufacturer-specified absolute accuracy of the infrared thermometer (±3.0 °C at 0–25 °C). Accordingly, the differences between individual time points and between the two transducers cannot be interpreted quantitatively. These results indicate only that no gross progressive increase in the indicated surface temperature was detected under the specific test conditions; they do not conclusively demonstrate long-term thermal or operational stability.

Overall, the APUT provides a feasible transducer architecture for reducing estimated component-level cost and device weight while retaining broadband, high-frequency ultrasound imaging capability. Conductive shielding partially reduced the measured noise associated with the polymer-housed APUT, although improvements in echo sensitivity, shielding and grounding design, fabrication reproducibility, and performance during prolonged operation remain necessary. With these improvements, together with standardized electromagnetic-compatibility testing and subsequent in vivo validation, this architecture may provide a basis for the future development of lightweight and low-cost ultrasound devices and may potentially serve as a component of wearable, portable, or disposable imaging systems.

## 5. Conclusions

In this study, a high-frequency PVDF ultrasound transducer incorporating aluminum electrodes and a 3D-printed polymer housing was developed and compared with a reference gold-electrode/metal-housing transducer. Under the cost assumptions specified in this study, the estimated component-level cost of the APUT was 6.74 USD, approximately 66% lower than that of the GMUT, while its total device weight was reduced from 11.3 to 1.6 g. The APUT retained a broadband acoustic response and high-frequency imaging capability, although it exhibited a lower echo amplitude and SNR than the GMUT. Its smaller measured axial and lateral FWHM values demonstrated fine spatial resolution under the investigated conditions, and the successful visualization of embedded targets in a layered ex vivo chicken-breast specimen supported its feasibility for biological-tissue imaging. Applying a conductive shielding layer reduced the RMS noise voltage of the APUT from 9.7 to 5.1 mV and increased its SNR from 11.6 to 17.1 dB, although its overall noise performance remained lower than that of the GMUT. During the 3-h pulsed-operation test, the APUT exhibited an initial capacitance increase of approximately 8.7%, followed by no clear progressive trend, while no gross progressive increase in the indicated surface temperature was detected for either transducer. Overall, these results support the feasibility of aluminum electrodes and 3D-printed polymer housings as lightweight and lower-cost material choices for compact PVDF ultrasound transducers. Further improvements in echo sensitivity, electromagnetic shielding, grounding, fabrication reproducibility, and performance during prolonged operation, together with standardized electromagnetic-compatibility testing and in vivo validation, will be required before practical or clinical implementation.

## Figures and Tables

**Table 1:** Comparison of the material costs and weights of the GMUT and APUT.

	GMUT			APUT		
Component	Unit Cost	Amount	Cost (USD)	Unit Cost	Amount	Cost (USD)
Electrode PVDF film	450 USD/sheet	9π mm^2^	0.88	250 USD/sheet	9π mm^2^	0.12
Backing layer	0.96 USD/g	0.34 g	0.33	0.96 USD/g	0.34 g	0.33
Coaxial connector	2.00 USD/ea	1 ea	2.00	2.00 USD/ea	1 ea	2.00
Housing	11.63 USD/ea	1 ea	11.63	4.22 USD/ea	1 ea	4.22
Ground electrode	4.73 USD/ea	1 ea	4.73	6.77 USD/g	0.01 g	0.07
Total		11.3 g	19.57 USD		1.6 g	6.74 USD

## Data Availability

Experimental data are available from the corresponding authors upon reasonable request.

## Supplementary Materials

The following supporting information can be downloaded at: https://www.mdpi.com/article/10.3390/bios16090455/s1, Figure S1: Pulse–echo responses of the (a) unshielded APUT, (b) shielded APUT, and (c) GMUT with a conventional metallic housing.
