# Supplementary material for: A Low-Cost, Lightweight High-Frequency Ultrasound Transducer with Aluminum Electrodes and 3D-Printed Polymer Housing
Source: Biosensors (Basel). 2026 Aug 22;16(9):455. doi: 10.3390/bios16090455 (PMC13604671; doi:10.3390/bios16090455)
Supplement: Supplementary file 1 [file biosensors-16-00455-s001.zip › biosensors-4452900-supplementary.pdf]

Supplementary Information for

A Low-cost, Lightweight High-Frequency Ultrasound Transducer with Aluminum Electrodes and 3D-Printed Polymer Housing

Hyungjung Kim[1], Woohyun Jin[1], Do-Kyung Kim[2], Jaewoo Kim[3,*] and Jeongwoo Park[1,4,5,6,*]

[1] Department of Biomedical Convergence Science and Technology, Kyungpook National University, Daegu 41566, Republic of Korea

[2] Department of Advanced AI Engineering, Kangwon National University, Samcheok 25913, Republic of Korea

[3] Department of Convergence IT Engineering, and Medical Device Innovation Center, Pohang University of Science and Technology (POSTECH), Pohang 37673, Republic of Korea

[4] Department of Advanced Bioconvergence, Kyungpook National University, Daegu 41566, Republic of Korea

[5] Department of Medical and Biological Engineering, Kyungpook National University, Daegu 41944, Republic of Korea

[6] Cell and Matrix Research Institute, Kyungpook National University, Daegu 41944, Republic of Korea

[*]Corresponding authors: Jeongwoo Park (jwpark@knu.ac.kr)

Jaewoo Kim (kimjaewoo@postech.ac.kr)

Contents

Supplementary Figures

Figure S1. Additional Pulse–Echo Comparison of the Unshielded APUT, Shielded APUT, and GMUT

Supplementary Figure S1. Additional Pulse–Echo Comparison of the Unshielded APUT, Shielded APUT, and GMUT

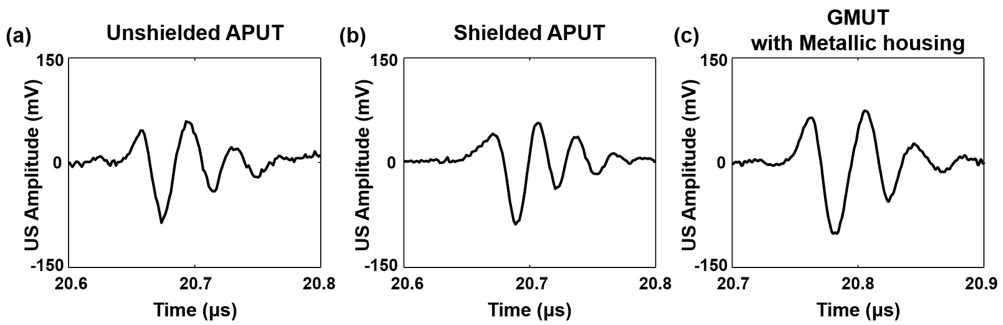

Figure S1. Pulse–echo responses of the (a) unshielded APUT, (b) shielded APUT, and (c) GMUT with a conventional metallic housing. All three configurations were evaluated within the same measurement session under nominally identical excitation and acquisition conditions.

An additional pulse–echo experiment was performed to compare the unshielded APUT, the same APUT after application of a conductive shielding layer, and the GMUT under nominally identical conditions within the same measurement session. The conductive shielding layer consisted of copper foil tape wrapped around the outer surface of the plastic housing without covering the active aperture. The copper foil layer was electrically connected to the ground terminal.

The RMS noise voltages of the unshielded APUT, shielded APUT, and GMUT were 9.7, 5.1, and 2.5 mV, respectively, and the corresponding SNR values were 11.6, 17.1, and 25.0 dB. The shielding treatment reduced the RMS noise voltage of the APUT by approximately 47% and increased its SNR by 5.5 dB, although the resulting noise performance did not reach that of the GMUT.

Because no controlled electromagnetic-interference source was applied and the transducers also differed in their electrode and grounding configurations, these results indicate a reduction in measured noise under the tested laboratory conditions rather than a quantitative measurement of housing shielding effectiveness.
